# Determinants of Drug Resistance in Previously-Treated Pulmonary Tuberculosis Patients Registered at a Chest Clinic in South Delhi, India

**DOI:** 10.7759/cureus.5541

**Published:** 2019-08-31

**Authors:** Manila Sharma, Neelam Roy, Rupsa Banerjee, Jugal Kishore, Ashok Jakhar

**Affiliations:** 1 Community Medicine, Vardhman Mahavir Medical College and Safdarjung Hospital, New Delhi, IND

**Keywords:** pulmonary tuberculosis, previously treated, drug resistance, factors associated, cross-sectional study, new delhi, india

## Abstract

Introduction

Drug-resistant tuberculosis (DR-TB) is a major concern to effective control of tuberculosis (TB) in India and the likelihood of drug resistance increases with repeated exposure to anti-TB drugs. India has emerged as one of the leading contributors of DR-TB in the world posing a major threat to TB control. In the current study, we aim to find the burden and factors associated with drug resistance in previously treated pulmonary TB patients.

Methods

A cross-sectional study was conducted among 230 previously treated pulmonary TB patients registered with Directly Observed Treatment, Short-course (DOTS) centers under Nehru Nagar Chest clinic in Delhi, India. The participants were selected consecutively as they registered with the chest clinic. A predesigned, pretested, semi-structured questionnaire in the Hindi language used to collect socio-demographic data and factors associated with the development of drug resistance. Physical examination of all the participants was done (height, weight, pallor). Data were analyzed using SPSS version 21. Binary logistic regression analysis was used to identify independent risk factors of drug resistance.

Results

Of 230 previously treated pulmonary TB patients, 80 (34.8% (95% CI:28.7-40.9%)) were drug-resistant. Age (p=0.021), ever consumption of alcohol (p= 0.001), pallor (p=0.06), BMI (p=0.028), fasting blood sugar (p=0.001), treatment failure (p=0.005) and the number of prior courses of anti-tuberculosis treatment (ATT) taken (p=0.004) were significantly associated with drug resistance. On applying binary logistic regression analysis, independently associated factors with drug resistance were ever consumption of alcohol, pallor, high fasting blood sugar level, previous treatment failure patients and the number of prior courses of ATT (p<0.05).

Conclusion

The findings of this study revealed that patients who had pallor, high fasting blood sugar, treatment failure and who had two or more prior courses of ATT were more likely to have DR-TB. Identifying the risk factors for drug-resistant TB is essential in facilitating the government to draw public health interventions. Further research is warranted to explore the causal associations.

## Introduction

Tuberculosis (TB), a major global health problem since ancient times, believed to have originated 150 million years ago. It has a major impact on a country’s social and economic productivity. Alongside (Human Immunodeficiency Virus) HIV, TB is the second most common cause of death worldwide [1]. According to the Global TB Report 2018 of the World Health Organization (WHO), 10 million new cases of TB developed in 2017 worldwide and India contributed 27% of the cases [2]. In 2017, TB caused an estimated 1.3 million deaths in HIV negative individuals plus an additional 300, 000 in HIV positive people. Mortality due to drug-resistant tuberculosis (DR-BT) was 410, 000 deaths approximately [3].

DR-TB has been a topic of growing interest in the last decade. Until 1994-1997 when the global project on anti-TB drug resistance surveillance was started by WHO and the International Union Against Tuberculosis and Lung Disease (IUAT-LD), the exact magnitude of TB burden was not known to the world [4]. DR-TB has emerged as a major setback to effective TB control globally. The threat being greater in countries like India, resource-limited developing countries. Many studies have reported poor treatment adherence, irregular treatment, long duration of treatment as the risk factors for the development of DR-TB.

During the last decade, there has been an increase in reported incidences of drug resistance in previously treated patients, particularly among those treated irregularly, or with incorrect regimens and doses. Some studies suggest that the most important risk factor for the development of DR-TB is the previous treatment of TB [5-9]. TB is a constantly changing disease as it varies with the population composition, cultural practices, and attitude of the people. Therefore, the research needs to be constantly updated to keep the scientific community and the health system of the country abreast with them, so that the government policies can keep up with the changing trend.

In Delhi, most of the studies were done to find out the pattern of drug resistance among new TB patients or Multi-Drug Resistant TB. The factors associated were not studied in detail among previously treated pulmonary TB patients. There was no focus on overall drug resistance. Identifying factors associated with the development of resistance and studying their interaction is important. Hence, in the current study, we plan to determine the burden of drug resistance and associated factors of drug-resistance in previously treated pulmonary TB patients. 

## Materials and methods

Study design and setting

This was a cross-sectional study conducted between November 2016 and April 2018 at the Directly Observed Treatment, Short-course (DOTS) centers under Nehru Nagar Chest Clinic in New Delhi, India. Pretesting, modification of the questionnaire, and groundwork for data collection was done by December 2016. Data collection was done for 13 months from January 2017 to January 2018. There were a total of 25 chest clinics in Delhi under the Revised National Tuberculosis Control Program (RNTCP). The current study was conducted in the Nehru Nagar chest clinic which was selected out of these 25 chest clinics by simple random sampling. There were 3 TB Units and 42 DOTS Centers under this Chest Clinic. A total of approximately 15,000 patients registered in the preceding year i.e. 2015 at Nehru Nagar Chest Clinic. Among these, there were about 3600 patients in previously treated including 2600 pulmonary TB cases. DR-TB registering with the clinic were approximately 480 annually. Previously treated pulmonary TB patients (Recurrent TB, Treatment after Failure, Treatment after Loss to Follow- Up) both sputum smear-positive and sputum smear-negative were included in the study. Serious/debilitated patients unable to give consent and interview were excluded from the study.

Sample size and sampling technique

The sample size was calculated by taking the prevalence of drug resistance in India among retreatment cases of pulmonary TB as 15% as per the Global TB report 2015 [10]. We took the confidence interval (CI) as 95%, power as 80%, absolute precision as 5% and calculated sample size using the formula 4pq/d^2^ [where p=prevalence taken; q= (1-p); d=precision]. After adding a 10% non-response rate to it, the minimum sample size came out to be 224, which was rounded off to 230. All the patients were consecutively included from the register until the sample size of 230 was achieved.

A pre-designed, pre-tested, semi-structured, interviewer-administered questionnaire in the Hindi language was used to interview the study participants to elicit the relevant information. The questionnaire included questions on socio-demographic characteristics including age, sex, place of residence. The socio-economic status of the study participants was determined by using the Modified BG Prasad’s socioeconomic status scale, 2016 [11]. Information related to TB including clinical presentation in the current episode of TB, information related to drug resistance from the records, factors associated with drug resistance including the history of previous treatment for TB, smoking, alcohol consumption, co-morbidities, etc. were collected. General physical examination including anthropometric assessment and local examination of the study participants were done. Body Mass Index (BMI) cut-offs for adults ( >18 years) used were according to WHO Asian BMI guidelines (BMI < 18.5 kg/m^2^: underweight; 18.5 - 22.9 kg/m^2^: normal; 23 - 24.9 kg/m^2^: overweight; ≥25 kg/m^2^: obese) [12]. For study participants ≤ 18 years, WHO percentile nomograms for males and females were used [13]. Data were collected at the DOTS centers when the patients came to collect their medication.

Definitions

All the operational definitions were as per the national guideline laid out in the RNTCP 2016. “Treatment after Failure” was defined as patients who had been previously treated for TB and whose treatment failed at the end of their most recent course of treatment. “Recurrent TB” was defined as a TB patient previously declared as treated (cured/treatment completed) and was subsequently found to be a microbiologically confirmed TB case. “Treatment after Loss to Follow Up” were patients who received anti-tuberculosis treatment (ATT) for one month or more and were declared lost to follow-up in their most recent course of treatment and subsequently found to be microbiologically confirmed cases of TB [14].

Statistical analysis

Data Entry was done on Microsoft Excel spreadsheet and data analysis was done using the licensed Statistical Package for Social Sciences (SPSS) v. 21. The data were summarized and presented in the form of tables and appropriate diagrams. The qualitative data were summarized as proportions and quantitative data as mean (standard deviation) or median (Inter-quartile range (IQR)). Qualitative data were analyzed using the Chi-Square/Fisher exact test while quantitative data by t-test. The level of significance was set at p<0.05. Odds ratio (OR) and 95% interval were calculated to assess the magnitude of the association between risk factors and DR-TB.

Ethics

Ethical clearance was obtained from the Institutional Ethics Committee of Vardhman Mahavir Medical College and Safdarjung Hospital, before data collection. Written informed consent was obtained from the study participants.

## Results

Socio-demographic data

A total of 230 respondents participated in the study. The age of the study participants ranged from 10-80 years with a median age of 30 years (IQR: 21-42 years). Most of the participants belonged to the age group of 16-30 years (103; 44.8%) while 14 (6.1%) participants were 15 years or less. There were 157 (68.3%) males and 73 (31.7%) females. The majority of the study participants were married (139; 60.4%), most were Hindu by religion (192; 83.5%). It was observed that 54 (24.3%) of the study participants had received education only till 5th grade while 51 (22.2%) were illiterate. Almost half of the study participants were unemployed 109 (47.3 %), 38 (16.5%) were students, 26 (11.3%) were homemakers, and 20 (8.6%) were drivers. Most of the study participants belonged to the middle (83; 36.1) and lower middle class (67; 29.1%) according to Modified BG Prasad’s socioeconomic status scale (2016). Majority of the study participants lived in an urban area (186; 80.9%) with only 44 (19.1%) residing in rural areas. Co-morbidities were present in 52 (22.6%) of the study participants. It was found 96 (41.7%) study participants had ever smoked, 85 (36.6%) had ever consumed smokeless tobacco while 89 (38.7%) had ever consumed alcohol. The sociodemographic characteristics of the patients are shown in Table 1.

**Table 1:** Sociodemographic characteristics of study participants (N=230) *Others include cook, receptionist, lab assistant, salesman, tech support, carpenter, plumber, etc., #Co-morbidities included Diabetes Mellitus, Hypertension, Asthma, HIV, Silicosis, Thalassemia, Hepatic Cancer and Polycythemia; HIV: Human Immunodeficiency Virus

Age (in completed years)	Number (%)
≤ 15	14 (6.1)
16-30	103 (44.8)
31-45	65 (28.3)
46-60	40 (17.4)
≥ 60	8 (3.5)
Gender	
Male	157 (68.3)
Female	73 (31.7)
Marital Status	
Married	139 (60.4)
Unmarried	79 (34.3)
Divorced/Separated/Widowed	12 (5.2)
Religion	
Hindu	192 (83.5)
Muslim	33 (14.3)
Sikh/Christian	5 (2.1)
Education	
Illiterate	51 (22.2)
Upto 5th grade	56 (24.3)
Upto 8th grade	42 (18.3)
Upto 10th grade	31 (13.5)
Upto 12th grade	26 (11.3)
College and above	24 (10.0)
Occupation	
Unemployed	109 (47.3)
Students	38 (16.5)
Homemaker	26 (11.3)
Construction Worker	20 (8.6)
Driver	10 (4.3)
Tailor	6 (2.6)
Shopkeeper/ Clerical	4 (1.7)
Others^*^	17 (7.4)
Socio-Economic Status	
Upper Class	17 (7.4)
Upper Middle	49 (21.3)
Middle	83 (36.1)
Lower middle	67 (29.1)
Lower	14 (6.1)
Patient Residence	
Urban	186 (80.9)
Rural	44 (19.1)
Co-Morbidities^#^	
Present	52 (22.6)
Absent	178 (77.4)
Ever Smoked	
Yes	96 (41.7)
No	134 (58.3)
Ever Consumed Alcohol	
Yes	89 (38.7)
No	141 (61.3)
Ever Consumed Smokeless Tobacco	
Yes	85 (36.6)
No	145 (63.4)

Clinical characteristics

Pallor was found to be present in 93 (40.4%) of the study participants, about three-fourths (172; 74.8%) of the study participants were underweight. The mean fasting blood sugar was 121.1 ±72.8 mg/dl. The majority of the study participants (142; 61.7%) had normal fasting blood sugar of less than 110 mg/dl, and 40 (17.4%) had blood glucose more than 126 mg/dl which was abnormal. The clinical characteristics of patients are shown in Table 2.

**Table 2:** Distribution of study participants according to clinical characteristics (N=230) *Categorization of BMI done as per WHO Asian cut-offs for adults and as per BMI nomogram for participants ≤ 18years; WHO: World Health Organisation, BMI: Body Mass Index, mg/dl: milligrams per decilitre

Clinical Characteristics	Number (%)
Pallor	
Absent	137 (59.6)
Present	93 (40.4)
Body Mass Index (BMI)^*^	
Underweight	172 (74.8)
Normal	52 (22.6)
Overweight	6 (2.6)
Fasting Blood Sugar Levels (mg/dl)	
< 110	142 (61.7)
110 – 125	48 (20.9)
≥126	40 (17.4)

Treatment history

Out of the total study participants, the majority were recurrent TB cases (128; 55.7%). More than three-fourths of the study participants had taken ATT once before the current episode (186; 80.9%), 29 (12.6%) study participants had taken two prior courses of ATT and 14 (6.1%) participants had taken 3 or more courses of ATT. More than half (138; 60.0%) of the study participants were taking regular treatment during the previous episode of TB while 92 (40.0%) took irregular treatment. The majority of the study participants had completed their last course of ATT (146, 63.5%) but more than 1/3rd (84, 36.5%) had not. The treatment history of TB patients is shown in Table 3.

**Table 3:** Distribution of study participants according to treatment history (N=230) TB: Tuberculosis, ATT: Anti-Tuberculosis Treatment

Type of Patient	Number (%)
Recurrent TB	128 (55.7)
Treatment After Loss to Follow Up	83 (36.0)
Treatment After Failure	19 (8.3)
Number of Prior Courses of ATT	
1	186 (80.9)
2	29 (12.6)
≥ 3	15 (6.5)
Treatment Compliance (in the previous episode of TB)	
Regular	138 (60.0)
Irregular	92 (40.0)
Treatment Completion (last course of ATT)	
Completed treatment	146 (63.5)
Incomplete treatment	84 (36.5)

Burden of drug resistance

**Figure 1:**
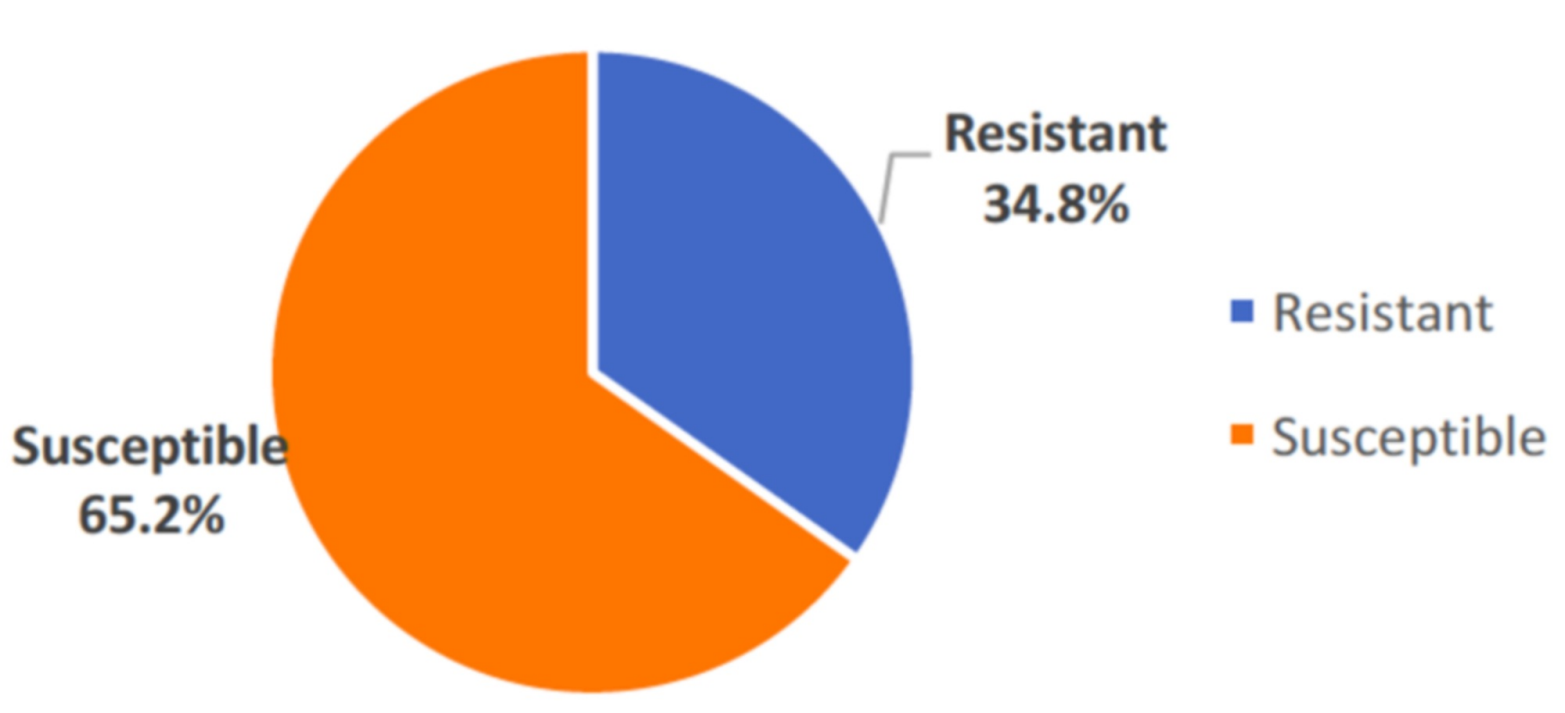
Distribution of study participants according to drug susceptibility (N=230)

Drug resistance was found in 80 i.e. 34.8% (CI: 28.7- 40.9%) of the study participants. The drug susceptibility of the patients is depicted in Figure 1.

Associated factors with drug-resistance

On applying appropriate tests of significance, drug resistance was found to be associated with age (p=0.021), ever consumption of alcohol (p= 0.001), pallor (p=0.06), BMI (p=0.028), fasting blood sugar (p=0.001), type of patient (p=0.005) and the number of prior ATT courses (p=0.004). Factors associated with drug resistance are shown in Table 4.

**Table 4:** Distribution of study participants according to the associated factor of drug resistance (N=230) *Chi-square test, #Fisher-Exact test; BMI: Body Mass Index, mg/dl: milligrams per decilitre, TB: Tuberculosis, ATT: Anti-Tuberculosis Treatment,

Variables	Drug Susceptibility			p-value
	Sensitive n (%)	Resistant n (%)	Total n (%)	
Age (in completed years)				
0-18	14 (43.8)	18 (56.3)	32 (100.0)	0.021^#^
19-60	131 (68.9)	59 (31.1)	190 (100.0)	
>60	5 (62.5)	3 (37.5)	8 (100.0)	
Sex				
Male	106 (67.5)	51 (32.5)	157 (100.0)	0.28^*^
Female	44 (60.3)	29 (39.7)	73 (100.0)	
Education				
Illiterate	38 (74.5)	13 (25.5)	51 (100.0)	0.377^*^
Upto 5th grade	38 (67.9)	18 (32.1)	56 (100.0)	
Upto 8th grade	25 (59.5)	17 (40.5)	42 (100.0)	
Upto 10th grade	17 (54.8)	14 (45.2)	31 (100.0)	
Upto 12th grade/Graduate	32 (64.0)	18 (36.0)	50 (100.0)	
Socioeconomic Status				
Upper Class	16 (94.1)	1 (5.9)	17 (100.0)	0.060^#^
Upper Middle	32 (65.3)	17 (34.7)	49 (100.0)	
Middle	52 (62.7)	31 (37.3)	83 (100.0)	
Lower Middle	39 (58.2)	28 (41.8)	67 (100.0)	
Lower	11 (78.6)	3 (21.4)	14 (100.0)	
Comorbidities				
Absent	115 (64.6)	63 (35.4)	178 (100.0)	0.719^*^
Present	35 (67.3)	17 (32.7)	52 (100.0)	
Substance Abuse				
Ever Smoked				
Yes	86 (64.2)	48 (35.8)	96 (100.0)	0.696^*^
No	64 (66.7)	32 (33.3)	134 (100.0)	
Ever Consumed Alcohol				
Yes	70 (78.7)	19 (21.3)	89 (100.0)	0.001^*^
No	80 (56.7)	61 (43.3)	141 (100.0)	
Ever Consumed Smokeless Tobacco				
Yes	55 (64.7)	30 (35.3)	85 (100.0)	0.901^*^
No	95 (65.5)	50 (34.5)	145 (100.0)	
Pallor				
Absent	99 (72.3)	38 (27.7)	137 (100.0)	0.006^*^
Present	51 (54.8)	42 (45.2)	93 (100.0)	
Body Mass Index (BMI)				
Underweight	104 (60.5)	68 (39.5)	172 (100.0)	0.028^*^
Normal	40 (76.9)	12 (23.1)	52 (100.0)	
Overweight	6 (100.0)	0 (0.0)	6 (100.0)	
Fasting Blood Sugar (in mg/dl)				
< 110	105 (73.9)	37 (26.1)	142 (100.0)	0.001^*^
110-125	22 (45.8)	26 (54.2)	48 (100.0)	
≥126	23 (57.5)	17 (42.5)	40 (100.0)	
Type of Patient				
Treatment after Failure	6 (31.6)	13 (68.4)	19 (100.0)	0.005^*^
Treatment After Loss to Follow Up	55 (66.3)	28 (33.7)	83 (100.0)	
Recurrent TB	89 (69.5)	39 (30.5)	128 (100.0)	
Number of Prior ATT Courses				
1	130 (69.9)	56 (30.1)	18 (100.0)	0.004^*^
2	15 (51.7)	14 (48.3)	29 (100.0)	
≥ 3	5 (33.3)	10 (66.7)	15 (100.0)	
Treatment Compliance				
Irregular	57 (62.0)	35 (38.0)	92 (100.0)	0.397^*^
Regular	93 (67.4)	45 (32.6)	138 (100.0)	

Predictors of drug resistance

Binary logistic regression analysis revealed the significantly associated factors with drug resistance: ever consumption of alcohol, pallor, fasting blood sugar level, type of patient, and the number of times prior ATT courses (p<0.05). The study participants who had never consumed alcohol had 2.8 times the risk of having drug resistance as compared to those who said they had consumed alcohol (OR= 2.8; 95% CI 1.49-5.40). The patients who had pallor had a 1.8 times higher risk of having drug resistance as compared to those who did not have any pallor. (OR=1.8; 95% CI 1.02-3.27). The study participants who had an abnormal fasting blood sugar (≥110 mg/dl) had a 2.3 times higher risk of having drug resistance as compared to those who had normal fasting blood sugar (<110 mg/dl) (OR=3.3; 95% CI 1.69-6.62). It was observed that study participants who were treated after failure had 4.08 times the risk of having drug resistance as compared to those were treated after loss to follow up or recurrent TB. (OR=4.08; 95% CI 1.42-11.70). Those study participants who had taken two or more prior courses of ATT had 2.4 times the risk of having drug resistance as compared to those who had taken it only once before (OR=2.4; 95% CI 1.19-5.21). The predictors of drug resistance are shown in Table 5.

**Table 5:** Predictors of drug resistance among study participants (N=230) OR: Odds Ratio, CI: Confidence Interval, mg/dl: milligrams per decilitre, TB: Tuberculosis, ATT: Anti-Tuberculosis Treatment

Factors	n (%)	Odds Ratio (OR)	95% CI of OR	p-value
Age (in years)				
≤ 18	32 (13.9)	1.2	0.37- 4.03	0.727
> 18	198 (86.1)	Reference		
Ever consumed alcohol				
Yes	141 (61.3)	Reference		
No	89 (38.7)	2.8	1.49 - 5.40	0.001
Pallor				
Absent	137 (59.6)	Reference		
Present	93 (40.4)	1.8	1.02-3.27	0.042
Body mass index				
Underweight	172 (74.8)	2.01	0.95 - 4.26	0.067
Normal/Overweight	58 (25.2)	Reference		
Fasting blood sugar (in mg/dl)				
< 110	142 (61.7)	Reference		
≥ 110	88 (38.3)	2.3	1.32 - 4.09	0.003
Type of patient				
Recurrent TB/ Loss to follow-up	211 (91.7)	Reference		
Treatment failure	19 (8.3)	4.08	1.42 -11.70	0.009
No. of Prior ATT courses				
1	186 (80.9)	Reference		
≥ 2	44 (19.1)	2.4	1.19 - 5.21	0.015

## Discussion

This study provides us with the burden of drug resistance in previously treated pulmonary TB patients. It also gives pertinent information on the various factors associated with drug resistance TB which can guide India in planning activities to tackle this growing problem namely the presence of pallor, fasting blood sugar ≥110 mg/dl, prior treatment failure, ≥ 2 prior courses of anti-tubercular treatment.

We found the burden of drug resistance among previously treated pulmonary TB patients to be 34.8% (95% CI: 28.7-40.9%). RNTCP National Drug resistance survey report 2017, reported the prevalence of any resistance in previously treated patients as 36.82% (Range 34.64%-39.04) [15]. As per some Indian studies, the prevalence of any drug resistance among previously treated TB patients was 37%-62.77% [6,16-17]. As per a few international studies, the prevalence of any drug resistance among previously treated TB patients was between 7.7%-81.7% [18-20].

In the present study, drug resistance was found to higher in patients who had never consumed alcohol. This was in contrast to a study by Fregona et al. (2016) in Brazil where chronic alcohol consumption was significantly associated with drug resistance [21]. Marahatta et al. in Nepal found no association between alcohol and DR [22]. Bhat et al. (2015) in a cross-sectional study in Madhya Pradesh reported a significant association between alcohol consumption and pulmonary TB (OR 3.2; 95% CI 480.8-2254.8; p = 0.009). This may be an erroneous association that needs further exploration [16].

Multiple treatments with ATT in their lifetime was found to be significantly associated with drug resistance wherein those who had taken ATT two or more times in their life had 2.4 times higher odds of having drug resistance than those who had only one prior course of ATT. Baya et al in a cross-sectional study conducted in Bamako, Mali on 214 presumed Multi-Drug Resistant Tuberculosis (MDR-TB) reported similar results, that those who had taken two courses of prior TB treatment (OR = 3.25, 95% CI: 1.44-7.30) were significantly associated with confirmed MDR-TB disease [23]. Multiple exposures of the bacteria to the drugs can lead them to gain adaptive mechanisms and become resistant to the action of the drugs or can be due to the natural selection of already resistant strains of the bacteria.

In the present study, treatment after failure patients were 4.08 times more likely to be drug-resistant as compared to recurrent TB patients and treatment after loss to follow up patients. Sharma et al. reported a higher proportion of drug resistance in defaulters among previously treated patients in Delhi [24]. Similar results to our study were reported by Baya et al. (OR = 3.82, 95% CI (1.87-7.79), p = 0.0002) [20]. This may be because in failure cases, the bacteria have a higher chance of developing resistance due to prolonged exposure to the drugs and also the bacteria are expected to be more resilient to the drugs which led to a failure of treatment in the first place.

One very interesting finding was that, drug resistance was found to be 1.8 times higher in study participants who had pallor on examination (45.2%) compared to those who did not have any pallor (38; 27.7%) and this difference was statistically significant (p<0.05). In a study by Nagu et al. (2014) in Tanzania, anemia prevalence was 86% in TB patients and these patients were 3 times more likely to have positive sputum smear at two months as compared to non-anemic patients (Relative Risk (RR) = 3.05; 95% CI 1.11-8.40, p = 0.03) and the risk for sputum positive smear results increased with severity of anemia (p-value for trend <0.0) [25]. The higher prevalence of drug resistance in anemia can be due to the weakened immune system. As has been studied before, delayed sputum smear conversion is a risk factor for both TB treatment failure and drug-resistant TB emergence [26]. In another study from Taiwan, among 34 patients who had delayed sputum conversion, 24 (70.6%) were found to have Isoniazid (INH) resistant strains of TB microbes [27]. In India, routine testing of hemoglobin is not done for all TB patients registering with the DOTS center and reserved only for serious cases. The program needs to include routine estimation of Hb and timely treatment of anemics for a better outcome as early detection and treatment are key. To understand better, the direct association between anemia and drug resistance needs to be explored further.

Participants with blood sugar above the cut-off level of 110 mg/dl had 2.3 times the odds of being drug-resistant compared to those with sugar level < 110 mg/dl. In a study by Fisher-Hoch et al (2008) in Texas and Mexico, MDR-TB was significantly associated with Type 2 Diabetes Mellitus (T2DM) (OR 2.1 95% CI 1.1-4.2) (OR 1.80 95% CI 1.1-2.9), respectively. In Texas, the majority of the T2DM patients with MDR-TB were resistant at their first culture at the time of diagnosis [28]. This may be explained by the impaired immunity due to high blood sugar, rendering them susceptibility to infection with resistant strains. This requires strict blood sugar monitoring for all TB patients and early control of the same.

Limitations: This study has a few limitations. First, it was limited to only one district of Delhi. There is a need for bigger studies with a larger sample size to shed light on this issue of drug resistance. Secondly, the cross-sectional nature of our study does not allow us to make any conclusion regarding the causal nature of any of the determinants. Despite the limitations, this study provides crucial information about drug resistance in Delhi among previously treated patients. Very few studies have investigated drug resistance factors in previously treated patients.

## Conclusions

The present study found the burden of drug resistance in previously treated pulmonary TB patients to be 34.8% (95% CI: 28.7-40.9%). Various factors observed to be significantly associated with drug resistance: never consumption of alcohol, presence of pallor, underweight individuals, fasting blood sugar ≥110 mg/dl, treatment after failure patients, ≥ 2 prior courses of ATT. They pose an imminent threat to TB control in India. Aggressive efforts such as strengthening the laboratory capacity to ensure timely detection, treatment, and monitoring of hemoglobin and deranged blood sugar. Activities to ensure patients and their caretakers understand the consequences of irregular and incomplete treatment should be upscaled. Strategies to ensure treatment adherence and completion especially in children and the importance of a good diet to ensure normal BMI need to be intensified.

## Appendices

Abbreviations

ATT- Anti-Tuberculosis Treatment

BMI- Body Mass Index

CI-Confidence Interval

DR-TB- Drug-Resistant Tuberculosis

DOTS- Directly Observed Treatment, Short Course

HIV- Human Immunodeficiency Virus

IUAT-LD- International Union Against Tuberculosis and Lung Disease

IQR- Inter-Quartile Range

ICMR- Indian Council of Medical Research

INH-Isoniazid

MDR- Multidrug-Resistant

OR-Odds Ratio

RNTCP- Revised National Tuberculosis Control Program

RR- Relative Risk

SPSS-Statistical Package for Social Sciences

TB - Tuberculosis

T2DM- Type 2 Diabetes Mellitus

WHO- World Health Organisation
